# B Cell Immunity in Lung Transplant Rejection - Effector Mechanisms and Therapeutic Implications

**DOI:** 10.3389/fimmu.2022.845867

**Published:** 2022-03-07

**Authors:** Birte Ohm, Wolfgang Jungraithmayr

**Affiliations:** ^1^Department of Thoracic Surgery, Medical Center – University of Freiburg, Faculty of Medicine, University of Freiburg, Freiburg, Germany; ^2^Department of Thoracic Surgery, University Hospital Zurich, Zurich, Switzerland

**Keywords:** lung transplantation, B cell, antibody mediated allograft rejection, chronic lung allograft dysfunction (CLAD), donor-specific antibodies, allosensitization

## Abstract

Allograft rejection remains the major hurdle in lung transplantation despite modern immunosuppressive treatment. As part of the alloreactive process, B cells are increasingly recognized as modulators of alloimmunity and initiators of a donor-specific humoral response. In chronically rejected lung allografts, B cells contribute to the formation of tertiary lymphoid structures and promote local alloimmune responses. However, B cells are functionally heterogeneous and some B cell subsets may promote alloimmune tolerance. In this review, we describe the current understanding of B-cell-dependent mechanisms in pulmonary allograft rejection and highlight promising future strategies that employ B cell-targeted therapies.

## Introduction

Lung transplantation is the definite therapeutic option for patients suffering from end-stage lung disease. However, long-term post-transplant survival is limited to approximately 50% after five years due to chronic lung allograft dysfunction (CLAD) (1). Generally, allogeneic immune responses are the barrier to unlimited pulmonary graft acceptance. While T-cell-dependent acute graft rejection is today efficiently prevented by immunosuppression, antibody-mediated rejection (AMR), characterized by the presence of donor-specific antibodies (DSA), remains a poorly controlled risk factor for CLAD development (2). Recently, B cells gained increasing attention as key allogeneic immune effectors *via* antibody-dependent and -independent mechanisms. B cells produce DSA and autoantibodies against pulmonary self-antigens that have implications in both, AMR and CLAD pathogenesis (2). Apart from inducing humoral immune responses, B cells also act as antigen-presenting cells (APCs) aiding in T cell activation. In chronically rejected allografts, B cells are critical for lymphoid neogenesis and the formation of in-graft tertiary lymphoid organs (TLOs). The latter are believed to promote a local alloimmune response (3).

B cells are functionally heterogeneous and not all subsets contribute to inflammatory graft injury. For example, regulatory B cell populations (Bregs) are thought to be critical mediators of immune homeostasis and graft tolerance (4). B cell-targeted therapeutic approaches could thereby improve long-term outcomes after lung transplantation.

## Humoral Alloimmunity Induces Complement-Dependent and –Independent Graft Injury

Preformed antibodies can cause hyperacute rejection with pulmonary allografts developing severe hemorrhagic oedema and radiographic infiltrates in the immediate postoperative period (5). Preformed donor-specific antibodies (DSA) against donor-derived human leukocyte antigen (HLA) molecules can be present in recipients due to prior sensitization (e. g. blood transfusion or pregnancy) or develop *de novo* upon transplantation.

**Figure 1:**
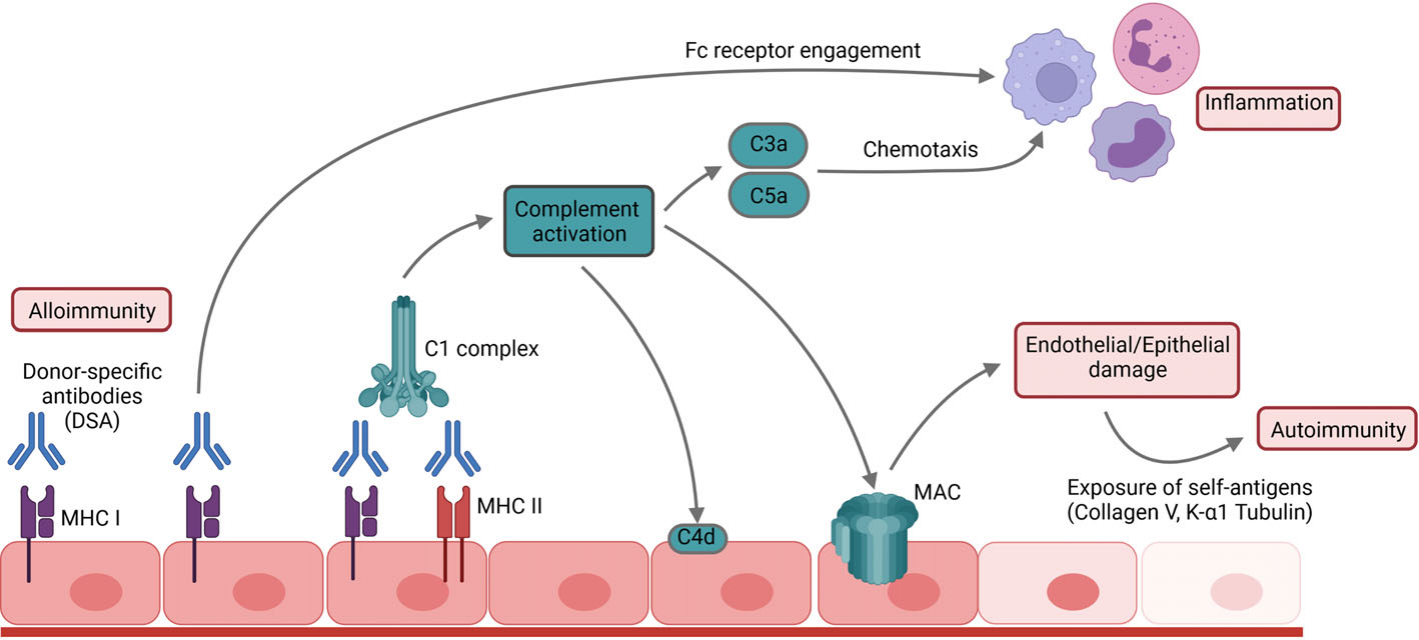
Mechanism of humoral allograft rejection. DSA and antibodies bind to their respective antigens and thus activate the complement cascade, promoting inflammation and leading to cellular damage by formation of the membrane attack complex (MAC). Subsequent exposure of pulmonary self-antigens such as Collagen V or K-α1 tubulin can further initiate autoimmune responses against the graft.

DSA can be directed against major histocompatibility complex (MHC) class I molecules, such as HLA-A, HLA-B and HLA-C or MHC class II molecules such as HLA-DQ, HLA-DR or HLA-DP (2). Cleary et al. demonstrated recently that the capillary endothelium is the primary target in anti-MHC I-antibody -mediated lung injury in a murine conditional knockout model (6). Notably, pulmonary endothelial cells not only carry MHC I, but also express MHC II antigens under inflammatory conditions (7, 8). The resulting immune complexes on the endothelial surface activate the classical complement pathway by engaging the C1 complex (**Figure 1**). Consequently, endothelial damage occurs due to the formation of the membrane attack complex (MAC) as the final effector of the complement cascade (9). Exposure of the basal membrane subsequently activates the coagulation cascade causing thrombosis, fibrinoid necrosis, hemorrhagic oedema and loss of graft function. During this process, pulmonary-self antigens are exposed and promote autoimmune responses and further graft damage (10, 11). The activation of the coagulation cascade can also further complement activation due to non-canonical cleavage of the C3 and C5 components (12). In addition, complement activation promotes inflammation by generating the anaphylatoxins C3a and C5a (9). However, not all DSA belong to complement-fixing immunoglobulin subclasses. Different mechanisms of complement-independent humoral allograft injury have been proposed including the release of growth factors that results in endothelial and smooth muscle cell proliferation or platelet activation (13, 14). Furthermore, DSA binding can promote cellular graft damage engaging the Fcγ receptors on natural killer cells, macrophages and neutrophils (15, 16). In lung transplantation, the presence of complement-binding IgG1- and IgG3-DSA is associated with worse post-transplant outcomes (17, 18).

Today, hyperacute rejection is sufficiently prevented by antigen avoidance due to prior detection of pre-formed panel reactive antibodies (PRA) in patients listed for transplantation and pre-transplant crossmatching. However, patients with high PRA titers have a decreased chance of transplantation, prolonged waiting times, and higher waitlist mortality (19). Management strategies for these patients vary significantly among institutions, some of them considering allosensitization even a contraindication to transplantation (20). Other centers employ desensitization techniques such as plasmapheresis or immune adsorption before transplantation with varying success (21). While these strategies may prevent hyperacute graft damage by transient reduction of circulating DSA measured by reduced MFI in DSA detection assays, they have no impact on the spectrum of PRA or the number of antibody-producing cells. As current allocation systems do not consider recipient sensitization, the question arises whether this criterion should be included to reduce waitlist mortality. However, consequentially higher rates of post-transplant complications might be observed. In 2022, the United States is expected to incorporate pre-transplant allosensitization in their allocation system (22).

## B Lymphocytes Initiate *De Novo* DSA Generation and Memory Responses Upon Transplantation

Upon transplantation, donor-derived antigen-presenting cells (APCs) and extracellular vesicles move to recipient secondary lymphoid organs (SLOs) and initiate an allospecific adaptive immune response. During this process, recipient T cells are activated by donor antigen by means of the *direct, indirect* or *semi-direct* allorecognition pathways. *Direct* allorecognition occurs when recipient T cells recognize intact donor MHC on donor APCs. Due to rapid elimination of donor-derived passenger leukocytes during the early post-transplant period, direct allorecognition is thought to be most relevant for acute rejection. However, the recent discovery of *semi-direct* allorecognition has challenged this paradigm. Here, recipient APCs acquire functional, intact donor MHC and can thus activate recipient T cells after the demise of passenger leukocytes. However, chronic allograft rejection is primarily a feature of *indirect* allorecognition in which recipient T cells recognize processed donor-derived antigen presented by recipient APCs (23). T cells activated by indirect allorecognition are subsequently able to translocate to the follicular border of the lymphoid follicle, acquire a T follicular helper cell (Tfh) phenotype and subsequently induce a B cellular response (24–26).

Naïve recipient B lymphocytes circulate to SLOs where they encounter donor antigen binding to their B cell receptor (BCR). Subsequently, the recipient B cell internalizes the donor antigen and processes it for presentation on MHC II. Such antigen-primed B cells express the G-protein coupled Epstein-Barr virus-induced molecule-2 (EBI2) on their surface and are mobilized to the border of the T cell zone (27). Here, they interact with their primed CD4^+^ T cell counterparts and subsequently undergo different fates: Firstly, short-lived antibody-producing plasma cells are generated. They reside in SLOs and rapidly produce low-affinity anti-donor antibodies. Secondly, B cells can differentiate to germinal center B cells (GC B cells), characterized by the expression of the B cell lymphoma 6 (BCL6) transcription factor. During the germinal center reaction, those B cells undergo clonal expansion, somatic hypermutation and affinity selection and thus generate a highly specific anti-donor response. The germinal center reaction thus results in the generation of memory B cells and long-lived plasma cells (LLPCs), both of which allow for a long-term upkeep of the donor-specific humoral immune response (28).

Memory B cells can be found in the spleen, lymph nodes, and peripheral blood. They reside in a quiescent state and await antigen re-challenge (29). Most memory B cells are class-switched and bind their specific antigen with a higher affinity than their naïve precursors (30). They retain all functional B cell properties and undergo activation, clonal expansion and germinal center reactions in a secondary immune response. Compared to naïve B cells, they show a faster kinetic due to their enhanced reactivation potential. They are thus essential players in allo-sensitization – allowing for rapid enhancement of DSA in pre-sensitized recipients (31). For example, memory B cells are generated during pregnancy and could thus induce a secondary immune response upon solid organ transplantation (32). However, the overall clinical significance of pregnancy-associated sensitization in allograft rejection remains unclear (33).

Apart from memory B cells allowing for the rapid generation of specific antibody upon antigen re-challenge, humoral memory is also mediated by constitutive antibody secretion from LLPCs. LLPCs are terminally differentiated cells and have lost both the ability to proliferate and most B cell-specific surface markers (34). They home to the bone marrow sinusoid niche where they persist for several years or even lifelong (35) and provide long-lasting and specific antibody secretion, even in the absence of their respective antigen (36). To date, there is only little understanding about the mechanisms leading to LLPC longevity. Even though LLPCs acquire an intrinsic transcriptional and metabolic profile that conditions them for long-term survival, continuous external signals from their survival niche are critical to maintain viability (35, 37). Therefore, the generation of LLPCs is conditional on their recruitment to the bone marrow mediated by the CXCL12-CXCR4 axis (38).

**Figure 2:**
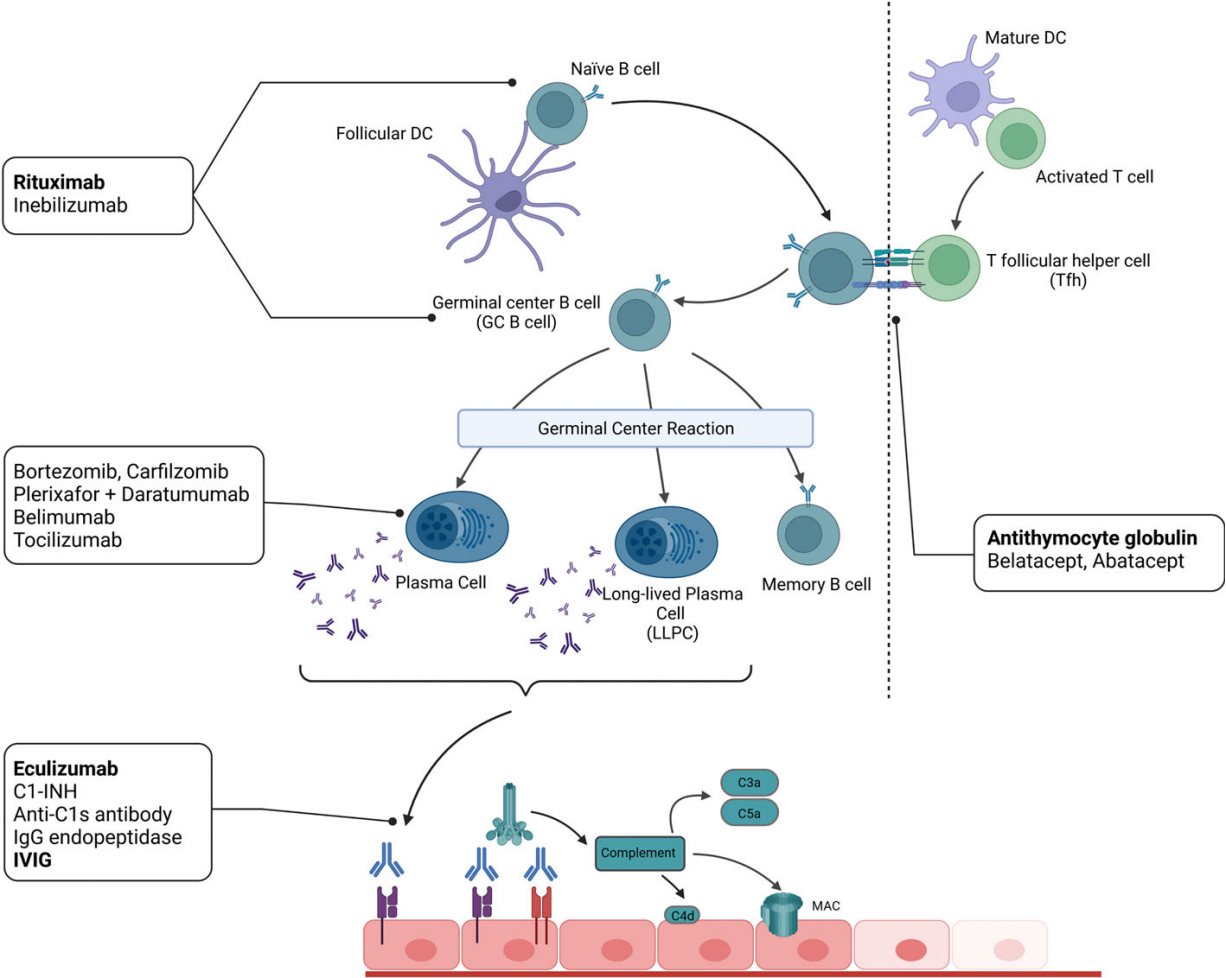
The B cell alloimmune response leads to the generation of graft-injuring alloantibodies and can be targeted therapeutically on different levels. Therapeutic agents in current clinical use in lung transplant recipients are depicted in bold.

By both reactivation of memory B cell responses and generation of LLPCs, lung transplant recipients develop a long-lasting humoral anti-donor immune response causing continuous inflammatory damage to the graft that ultimately leads to acute and chronic rejection (**Figure 2**).

## Antibody-Mediated Rejection Contributes to Chronic Rejection and Poor Outcome After Lung Transplantation

In 2016, the ISHLT consensus consortium defined for the first time the term antibody-mediated rejection (AMR) as a separate entity for pulmonary graft rejection and provided standardized diagnostic criteria (2). A definite diagnosis of AMR requires the diagnosis of clinical allograft dysfunction, the presence of DSA, a characteristic lung pathology, endothelial deposition of complement component C4d, and the exclusion of other causes of graft dysfunction.

Proof of C4d deposition is a controversial diagnostic criterion for pulmonary AMR (39). Most complement components show non-covalent binding to the endothelium and are thus rapidly inactivated by decay-accelerating factor (DAF) and CD59 on the endothelial surface. In contrast, C4d forms a covalent thioester bond making it resistant to shedding and thus detectable by immunohistochemistry. In pulmonary pathology, C4d deposition is not specific to AMR but is also observed during ischemia-reperfusion injury, acute cellular rejection and in the context of infection (40). In addition, not all cases of AMR show positive staining for C4d, giving rise to the descriptive term “C4d-negative” AMR. Whether this is a form of AMR caused by non-complement fixing antibodies or these observations owe to technical limitations of staining practices remains unclear (39).

Summarizing these aspects, AMR is a complex pathologic and clinical process that ultimately leads to acute and chronic loss of graft function. Clinically, AMR-related graft dysfunction can range from fulminant respiratory failure to subclinical dysfunction detected only by surveillance lung function testing (2). As the diagnosis of AMR is associated with increased presence of chronic lung allograft dysfunction (CLAD) (41), it seems likely that continuous humoral graft damage significantly contributes to chronic rejection. In support of this, the application of anti-donor MHC antibodies histologically results in obliterative bronchiolitis (OB) in murine studies (42). In lung transplant recipients with high DSA titers, restrictive allograft syndrome (RAS) is the more common CLAD subtype and often results in rapid and fatal graft failure (43). However, CLAD does also occur in the absence of detectable circulating DSA, and these cases cannot be histologically or clinically distinguished from those with measurable serum DSA. In some of these patients with missing serum DSA, the presence of intragraft DSA can be demonstrated (44, 45). These studies corroborate the importance of the humoral response in RAS as this CLAD phenotype was associated with higher in-graft DSA titers. The reason for the discrepancies between serum and intragraft DSA remains elusive (45). Firstly, they could be attributed to a strong intragraft binding of DSA, and secondly, some authors assign intragraft DSA to a local alloantibody production within the graft.

## Infiltrating B Cells May Promote Intragraft Alloimmune Reactions During Chronic Rejection

In chronically rejected allografts, infiltrating lymphocytes form cell clusters reminiscent of classical lymphoid follicles (46). This process is termed lymphoid neogenesis and is not only observed in chronically rejected solid organ grafts but also in chronic infection, autoimmune disease, and cancer (47). Generally, these tertiary lymphoid organs (TLOs) are formed when a persistently activated immune system is unable to eradicate its target antigen (48). In chronically rejected lung allografts, TLOs are typically found in patients suffering from restrictive allograft syndrome (RAS) (49). Here, the number of TLOs correlates with a lower life expectancy – indicating a deleterious role for TLOs. Even though the regulation of humoral responses upon transplantation is generally thought to occur peripherally and depend on antigen presentation in secondary lymphoid organs, TLOs are believed to elicit alloimmune responses by similar mechanisms (**Figure 2**) (50). Compared to secondary lymphoid organs, these processes occur directly within the graft in a microenvironment of abundant alloantigen and self-antigens released upon tissue damage (3). Furthermore, TLOs show restricted lymphatic drainage and are thus believed to trap antigen and initiate a pronounced local immune response (51). As a result, graft-damaging DSA could be generated within the transplant lung.

B cells are critical for maintaining lymphoid structures as B cell-deficient mice show disorganized lymphoid tissues (52). In a murine model of orthotopic single lung transplantation, Smirnova et al. recently demonstrated that the depletion of B cells abrogates TLO formation in pulmonary allografts. In addition, B cell depletion diminished the fibrous tissue remodeling that is characteristic for chronic rejection in this experimental model (53). Inhibition of EBI2 which is known for orchestrating B cell positioning in lymphoid structures, resulted in equal observations (53). Watanabe et al. demonstrated furthermore that B cell depletion diminished humoral immune responses against allo- and autoantigens, TLO formation and fibrosis in a murine orthotopic lung transplantation model of RAS using a minor-mismatched strain combination (54). They demonstrated that B cell depletion only had a minor influence on in-graft T cell composition - pointing towards an even more decisive role for B cells in the development of CLAD lesions (54). However, whether TLOs are a causative agent in CLAD or merely a byproduct of the immune response remains unclear. To date, adequate experimental models that can specifically inhibit lymphoid neogenesis without further targeting recipient immunity are missing. Thus far, it can also be proposed that the formation of TLOs in chronically rejected grafts is a non-specific response to the local inflammatory milieu (3).

## Alloimmunity-Induced Autoimmunity Through Breaking B Cell Self-Tolerance

Non-DSA humoral immune responses have gained interest in graft rejection (55). In principle, all human solid organ allograft recipients have autoantibodies. These antibodies target a wide variety of antigens which may be both ubiquitously present or organ-specifically expressed. In lung transplant recipients, autoantibodies that target fibrillar collagen V (ColV) and K-alpha tubulin (KαT) are associated with increased rates of CLAD (56). These autoantibodies either exist prior to transplantation or can be generated *de novo*. Their contribution to graft damage remains elusive as they are often accompanied by the presence of DSA (56). It is likely that the alloimmune response facilitates transplant-associated autoimmunity, as the majority of autoimmune target antigens are located in the intracellular compartment and are not expressed as surface molecules. However, antigens translocate to the cell surface upon apoptotic triggers such as previous alloimmune damage. In fact, transplant-associated autoantibody predominantly targets the allograft rather than native organs (57).

The development of autoimmune responses requires the breaking of B cell self-tolerance. Even in healthy individuals, clonal deletion and receptor editing in the bone marrow fail to eliminate all autoreactive cells and allow some self-reactive transitional B cells to enter the circulation. Upon antigen encounter, these cells acquire an anergic state that is characterized by limited B cell receptor signal transduction, a reduction of surface IgM and the exclusion from the B cell follicle (58). However, B cell anergy can be broken by CD4^+^ T cell help (59). Upon transplantation, the encounter of passenger donor CD4^+^ T cells could trigger autoimmunity by breaking B cell anergy (60). This may prime self-reactive B cells to interact with recipient-derived Tfh cells while subsequently undergoing a germinal center response.

Most likely, intra-graft TLOs contribute significantly to alloimmunity-induced autoimmunity as the abundance of self-antigens within the damaged graft may facilitate the selection of high-affinity mutants during the germinal center response. This way, the plasma cell output is shaped according to locally expressed autoantigen. In autoreactive germinal centers, epitope diversification may spread the autoreactive response to other autoantigens (61). In support of this, Bharat et al. demonstrated that anti-KαT-autoantibody is generated after the application of anti-ColV antibody in a murine model of unilateral lung transplantation (62).

The germinal center response further enhances the number of autoreactive B cells within the allograft. Here, these B cells function as APCs and contribute to graft damage by priming T effector cells (63). Even though antigen presentation by B cells requires the specific engagement of their B cell receptor, they could contribute to the alloimmune response by the uptake of bystander antigen within the graft (64).

## Polyreactive Natural Antibodies Putatively Participate in Lung Allograft Rejection

Polyreactive natural antibodies (nAbs) were first identified in the 1960s and play a decisive role in tissue homeostasis and innate immunity. Most nAbs originate from innate-like B1 B cells that primarily reside in the pleural and peritoneal cavities. However, marginal zone B cells and bone marrow precursors also contribute to nAb generation. nAbs include IgM, IgG and IgA isotypes with both germline and somatically mutated sequences (65).

nAbs are known to bind both apoptotic cell structures and pathogeneic antigen, and they control damage from oxidative stress. Similar to specific antibodies, nAbs can activate the classical complement pathway and engage Fc receptors (66, 67). With regard to transplantation, nAbs react to AB0 blood group antigens and xeno-antigens and are thus responsible for hyperacute rejection in AB0-incompatible- and xeno-transplantation (68). However, their role in allotransplantation is less clear.

Due to their broad binding profile, nAbs are difficult to evaluate and not readily distinguishable from specific antibodies. For example, polyreactive nAbs account in part for serum reactivity to HLA in Luminex assays (69). It is therefore difficult to study the specific role of natural antibodies.

Upon transplantation, nAbs may bind to endothelial neo-antigens exposed upon ischemia-reperfusion injury and recognize the danger-associated molecular patterns (DAMPs) in damaged allografts. In kidney transplantation, the presence of polyreactive nAbs is associated with AMR and graft loss (66, 70). While most nAbs belong to the IgM isotype in steady-state conditions, IgG nAbs dominate in kidney transplant recipients (71). I seems possible that innate-like B cells can undergo class switching following antigen encounter.

To our knowledge, there is only one study that evaluates the role of polyreactive nAbs in lung transplantation: in contrast to observations made in kidney transplantation, Budding et al. reported that elevated polyreactive nAbs against apoptotic cells in patients with end-stage lung disease do not show any correlation with the presence of acute or chronic allograft rejection (72).

Taken together, natural antibodies may play a role in allograft rejection by recognizing a broad variety of self- and non-self antigens. However, natural antibody responses are difficult to evaluate and their specific contribution to allograft damage remains largely unclear.

## B Cellular Antigen Presentation Contributes to Allograft Rejection

Even though antibody-mediated mechanisms significantly contribute to chronic allograft rejection, Zeng et al. demonstrated that B cells are able to promote chronic rejection independently of antibody production using a murine model of cardiac transplantation (63): while B cell-depleted animals were protected from it, mice lacking circulating antibodies developed chronic allograft vasculopathy. Upon adoptive transfer of B lymphocytes without the ability to produce antibodies, B cell-depleted animals developed chronic rejection (63). These observations may be attributed to the role of B cells as antigen-presenting cells and suggest that they are necessary for optimal priming of alloreactive T cells (73).

B cells are only able to present antigen *via* the indirect allorecognition pathway. Upon alloantigen engagement, they internalize the specific antigen and process it for presentation on MHC II (74). To our knowledge, MHC cross-dressing and therefore presentation for semi-direct allorecognition has not yet been described in B lymphocytes. Recipient B cells therefore exclusively engage with recipient CD4^+^ T cells. As described above, such an encounter is crucial for the germinal center response. Apart from this interaction with Tfh cells, it remains unclear whether B cells can function as APCs to other CD4^+^ effector or regulatory T cells in allogeneic transplantation (74).

## Regulatory B Cell Subsets Mediate Graft Tolerance

Apart from their detrimental role in allograft rejection, specialized B lymphocyte subsets have been implicated in promoting graft tolerance. These are immunosuppressive B lymphocytes that are collectively termed regulatory B cells (Bregs) and comprise a heterogeneous population of various stages of B cell maturity. Initially defined by the ability to produce interleukin-10 (IL-10), it is now accepted that Bregs also utilize other immunoregulatory cytokines and cell surface receptors (75). To date, characterizing Bregs remains challenging as they lack specific phenotypic or transcriptional markers. Insights into the development of Bregs is therefore limited (76). Whether these cells comprise a separate lineage or if B cells of different states of maturity can acquire regulatory properties under certain conditions is currently unclear (75).

Even though true transplant tolerance in the presence of generalized immunocompetence is not achievable in humans (77), operational tolerance is observed in liver and kidney transplant recipients, demonstrating stable graft function in the absence of maintenance immunosuppression. In these patients, B lymphocyte-related gene signatures have been characterized as biomarkers for operational tolerance (78). Tolerant recipients show reduced DSA titers and decreased numbers of plasma cells, as well as increased numbers of naïve B cells and IL-10^+^ Bregs in their peripheral blood (79). To date, operational tolerance has not been reported in lung transplantation. However, increased peripheral numbers of IL-10^+^CD19^+^ CD24^high^CD38^high^ transitional Bregs in lung transplant recipients were shown to be associated with a decreased risk for chronic rejection (80). These transitional Bregs have been defined to secrete the highest amount of IL-10 upon CD40 stimulation compared to other peripheral B cell subsets (75). In the murine CLAD model of heterotopic tracheal transplantation, an intense infiltration of transitional Bregs upon rapamycin treatment resulted in the prevention of fibrotic airway obliteration (81). These observations suggest that certain B cells mediate tolerogenic signals in lung transplantation and could therefore serve as innovative therapeutic targets in transplant-related diseases.

**Figure 3:**
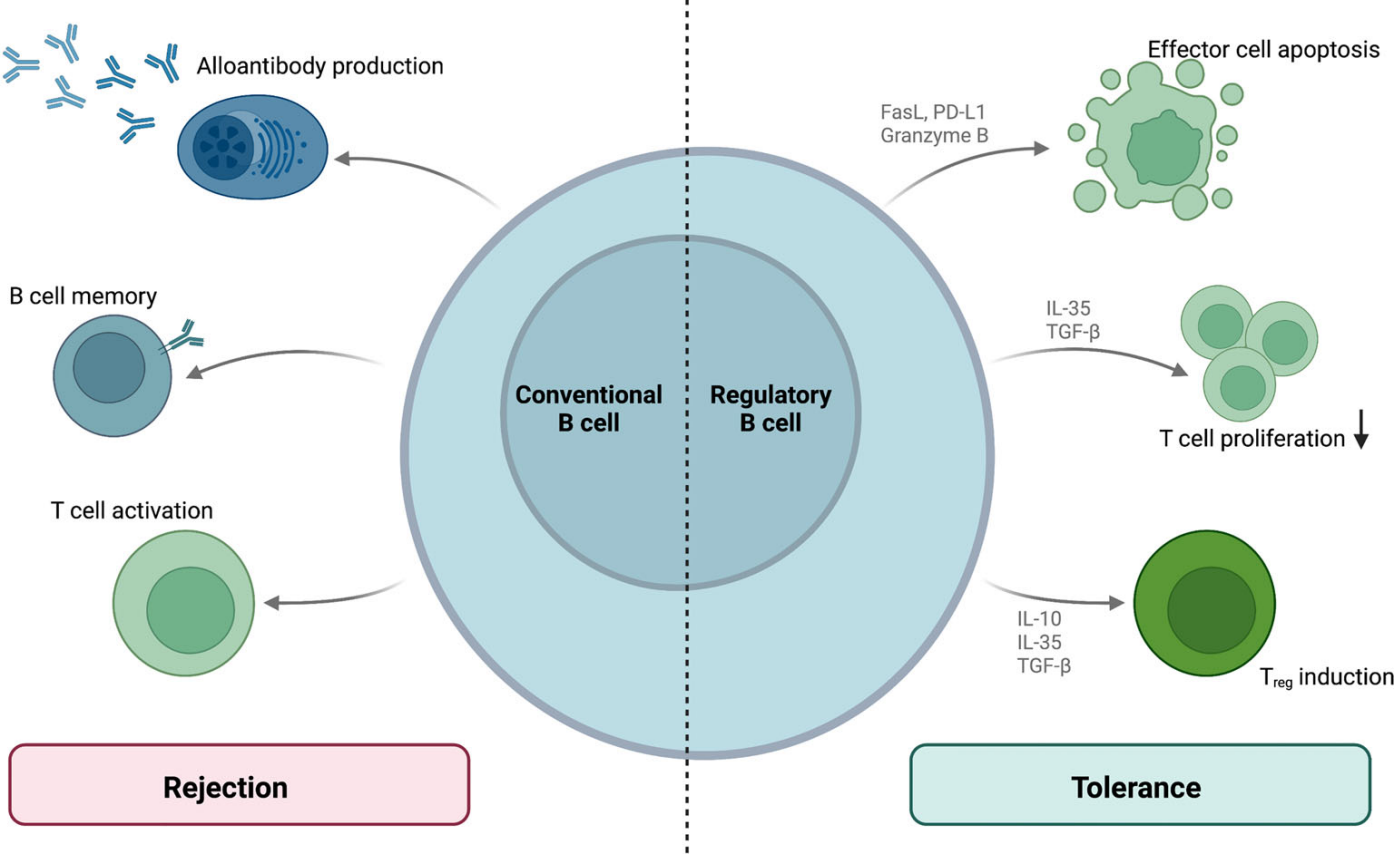
B cellular contribution to allograft rejection and tolerance. While conventional B cells mediate rejection by means of alloantibody production, memory responses and T cell activation, regulatory B cell subsets mediate allograft tolerance. These Bregs regulate T cell functions *via* surface molecules and cytokine production.

Mechanistically, Bregs target immune effector cells by both cytokine secretion and ligand-receptor interactions. Even though the nature of Breg interaction with other immune effectors *in vivo* remains unclear, surface expression of CD40 and MHC II suggests that Bregs interact with T lymphocytes and can therefore modulate T-cell-dependent alloimmunity (82). Engagement of Breg surface Fas ligand (FasL) or PD-L1 by their respective T cellular receptors Fas and PD-1 can induce apoptosis in T effector cells (83, 84). Bregs also indirectly modulate T cell function secreting the anti-inflammatory cytokines IL-10, IL-35 and transforming growth factor β (TGF-β) as well as granzyme B (82). As an anti-inflammatory member of the IL-12 cytokine family, IL-35 has been demonstrated to suppress T cell proliferation and boost the conversion of naïve T cells to IL-35^+^ regulatory T cells (Tregs) (85). TGF-β can support the generation of Tregs and inhibits effector lymphocyte proliferation (82). Furthermore, granzyme B can modulate effector lymphocyte populations by the induction of apoptosis (86). IL-10 - secreting Bregs (also termed B10 cells) also induce the differentiation and expansion of immunosuppressive regulatory T cells (Tregs) (87) and could thus promote graft tolerance. The tolerogenic properties of regulatory B cells are summarized and contrasted to conventional B cell-mediated rejection in **Figure 3**.

Taken together, some B lymphocyte subsets can mediate tolerogenic signals regulating T cell functions. Their immunomodulatory function should be considered when applying B cell-targeted therapy in the context of transplantation, as B cell depletion could thus increase the risk of cellular rejection (88).

## Strategies for AMR Treatment and DSA Reduction

Even though some may argue that T-cell-focused immunosuppression should curb B cell responses *via* Tfh cell inhibition, routine post-transplant immunosuppressive regimens fail to control AMR and chronic rejection after lung transplantation. Desensitization and current treatment strategies for pulmonary AMR have been adapted from experiences in other solid organ grafts. The treatment aims to diminish circulating DSA, limit complement activation or tone down B cell activity. **Figure 2** provides an overview of the strategies for AMR treatment and DSA reduction.

Plasmapheresis and immunoadsorption are often employed to reduce circulating DSA in highly sensitized recipients (89). While plasmapheresis separates plasma from whole blood, immunoadsorption specifically removes target antibodies from the circulation. However, the rates of DSA reduction are variable, and long-term desensitization cannot be achieved by plasmapheresis alone as antibody production is not suppressed. Indeed, rebound phenomena with heightened antibody production can occur after plasmapheresis (90). Therefore, plasmapheresis should always be used in combination with other therapeutic approaches.

As previously described, antibody-mediated complement activation significantly contributes to humoral graft damage. In addition, complement indirectly drives AMR development as opsonization (e.g. by C3d) is needed for antigen uptake into B cells. Upon BCR engagement, complement receptors act as B-cell coreceptors.

Eculizumab is an inhibitor of the complement component C5 routinely used in paroxysmal nocturnal hemoglobinuria (PNH) and atypical hemolytic syndrome (aHUS). In lung transplantation, the use of eculizumab has been employed in combination therapy for AMR (91) and hyperacute rejection (92). Inhibiting C5 cleavage to C5a and C5b, eculizumab can prevent graft damage by impeding MAC formation. However, eculizumab treatment not only limits activation of the classical pathway responsible for graft damage but also affects the alternative complement pathway central to pathogen clearance. Therefore, caution should especially be taken in transplant recipients regarding infectious complications during eculizumab treatment.

C1 esterase inhibitor (C1-INH) targets the classical pathway by blocking the proteolytic activity of C1r and subsequent cleavage of C2 and C4 components. Furthermore, C1-INH has inhibitory effects on the lectin pathway of complement activation, coagulation, kallikrein and kinin systems. In lung transplantation, C1-INH can effectively prevent reperfusion injury and primary graft dysfunction in experimental settings. In human pulmonary AMR, rapid clinical and radiologic improvements upon C1-INH treatment have been reported (93). The C1 complex can also be explicitly targeted by Anti-C1s monoclonal antibody which inhibits complement activation both *in vitro* (94) and *in vivo* (95) and reduces C4d deposition in renal AMR (96).

While these strategies reduce antibody-induced complement-mediated damage, they cannot achieve a reduction of antibody titers nor limit complement-independent graft injury. In this regard, IgG endopeptidase cleaves the Fc fragments from human IgG and thus renders them incapable of both complement activation and induction of antibody-dependent cellular cytotoxicity. In kidney transplantation, IgG endopeptidase treatment successfully reduced DSA and allowed successful HLA-incompatible transplantation in highly sensitized recipients (97). As of yet, a use in lung transplantation has not been reported. Even though IgG endopeptidase can reduce DSA titers, it exerts an unspecific effect on all IgG limited to a time period of approximately two weeks. Therefore, its use might not be feasible for preventing chronic humoral alloimmune responses.

Intravenous Immunoglobulin (IVIG) treatment is a commonly used immunosuppressive technique in autoimmune conditions (98) and is routinely employed in desensitization and AMR treatment. However, its precise mechanism of action remains unknown and IVIG might have a broad spectrum of effects on the immune response. For instance, IVIG treatment is believed to neutralize circulating DSA, inhibit complement activation and engage Fc receptors on immune effector cells (99). In lung transplantation, IVIG treatment alone does not sufficiently influence the humoral response (100) and is therefore often employed in combination with other agents (101).

To suppress antibody production, different therapeutics targeting the B cell response are available. Rituximab is a chimeric monoclonal anti-CD20 antibody inducing apoptosis in mature B cells. However, plasma cells lack CD20 surface expression and thus are not targeted by rituximab. Therefore, a reduction of antibody titers is observed with a delay of three months after the natural demise of short-lived plasma cells (102). In addition, LLPCs also lack CD20 expression, and thus rituximab cannot entirely abrogate the humoral response. In sensitized lung transplant recipients, rituximab is therefore often combined with antibody reduction strategies. In a prospective study, rituximab safely contributed to DSA reduction in combination with IVIG (103).

The anti-CD19 antibody inebilizumab can target a broader range of B lymphocytes as CD19 is conserved in plasma cells. In neuromyelitis optica, inebilizumab efficiently reduces pathologic antibody titers (104) and it is currently under clinical investigation for desensitization prior to renal transplantation (ClinicalTrials.gov Identifier: NCT04174677). However, evidence suggests that inebilizumab may not target LLPCs as these cells are not effectively targeted by CD19-directed CAR T-cell therapy (105).

When aiming to inhibit antibody production, plasma cells ought to be the primary therapeutic target. Proteasome inhibitors such as bortezomib and carfilzomib are small molecules first approved for use in multiple myeloma that deplete plasma cells by inducing apoptosis. In lung transplantation, proteasome inhibitors are used within multimodal concepts for desensitization and treatment of AMR (106, 107). However, beneficial long-term effects cannot be achieved with proteasome inhibitors. *In vitro*, they have been demonstrated to induce apoptosis in memory B cells. Thus, their lack of long-term efficiency can probably be attributed to the persistent activity of LLPCs in the bone marrow. Of note, bortezomib treatment is limited by cumulative dose-related toxicity and can have severe toxic pulmonary effects (108). Thus, caution should be taken when applying it to lung transplant recipients.

Therapeutically targeting LLPCs in the bone marrow niche is a significant hurdle to inhibiting alloantibody production. LLPCs evade B cell depletion strategies by altered expression of B cell markers and their upkeep is independent of T cell help. Plerixafor is a novel CXCR4 chemokine antagonist used to mobilize hematopoietic stem cells from the bone marrow (109). As LLPC homing is CXCR4-dependent, plerixafor could also achieve their mobilization (110). In murine studies, plerixafor treatment primarily mobilized splenic plasma cells (111). In a non-human primate model of sensitized renal transplantation, combining plerixafor with the plasma cell-directed anti-CD38 antibody daratumumab resulted in a reduction of DSA and prolonged graft survival (112). However, daratumumab treatment was associated with DSA rebound and robust T cell-mediated rejection (112). In clinical use, authors report on cases of successful employment of daratumumab for desensitization and AMR treatment (112). Currently, the safety of anti-CD38 antibody treatment for desensitization before kidney transplantation is evaluated in a phase Ib/II study (ClinicalTrials.gov Identifier: NCT04294459).

Belimumab is a monoclonal antibody targeting the cytokine B lymphocyte stimulator (BLyS) critical for B lymphocyte survival. As such, it has been proposed to limit plasma cell survival and could thus reduce humoral alloimmunity (113). In animal studies, BLyS inhibition resulted in DSA reduction and prolonged graft survival (114, 115). The use of belimumab has been evaluated in human kidney transplantation, demonstrating a good safety profile. However, authors did not find a significant influence on B cell populations (116). Use of belimumab in lung transplantation has not been reported to date.

The anti-IL-6-receptor antibody tocilizumab licensed for use in rheumatoid arthritis can have beneficial effects on B cell development in the context of transplantation (117). IL-6 is an essential cytokine for plasma cell differentiation, and IL-6-deficient mice present a reduction in IgG production (118, 119). In the context of lung transplantation, increased IL-6 is associated with primary graft dysfunction (120). In CLAD, the use of tocilizumab resulted in slight improvements in spirometry and DSA reduction (121).

Antithymocyte globulin (ATG) consists of polyclonal antibodies generated by applying human thymic tissue lysates to animals. Thus, ATG is directed against a wide variety of lymphocyte surface markers, including B cells. However, ATG more reliably affects T cells, and it is often reported that B cells are the predominating cell type persistent after treatment. Thus, ATG does not inhibit antibody production. However, the use of ATG in lung transplantation has been reported in various stages of graft damage and can ameliorate acute (122) and chronic graft rejection (123). ATG may convey protective effects by interfering with T cell support for B cell reactions.

Costimulation can be targeted therapeutically by the fusion proteins belatacept and abatacept consisting of human IgG1 Fc fragments and the extracellular CTLA-4 domain. By competitive binding to CD80 and CD86, these molecules prevent CD28 costimulation and, consequently, the activation of naïve T cells (124). These agents are approved for second-line maintenance immunosuppression in kidney transplantation, substituting calcineurin inhibitors. Evidence suggests that belatacept limits *de novo* DSA production (125). In lung transplantation, successful use of belatacept in maintenance immunosuppression has been reported (126) and its use is currently being formally evaluated (ClinicalTrials.gov Identifier: NCT03388008).

## Summary and Conclusion

B lymphocytes significantly contribute to the alloimmune response by antibody production and potentially by the modulation of the T cell response *via* antigen-presentation and cytokine secretion. Mainly, humoral responses cause hyperacute rejection and AMR. Clinically, sensitized lung transplant candidates face higher waitlist mortality and a reduced likelihood to be transplanted. Post-transplantation, antibody-mediated rejection can lead to a rapid loss of graft function and is associated with CLAD development. In chronic rejection, B lymphocytes initiate lymphoid neogenesis associated with a pronounced local immune response and a rapid functional decline – especially observed in the RAS phenotype. Thus, B cellular responses pose a significant hurdle to long-term pulmonary graft survival. Several therapeutic strategies aiming to tone down B cell responses and thus reduce circulating DSA are employed in the clinic. However, their success is limited. In clinical use, a combination therapy is recommended as no single agent can sufficiently diminish the B cell response alone. Multiple substances are necessary to target all, antibody-mediated graft injury, antibody production and plasma cell differentiation. Currently, several novel B cell-targeted therapeutic agents are under evaluation for kidney or lung transplantation. However, these immunosuppressants do not only inhibit the pathological immune reactions after transplantation but they also influence lymphocyte subsets with regulatory properties. Immunoregulatory B cells remain poorly characterized to date due to a lack of specific markers. Thus, selective inhibition of alloreactive B cells preserving Breg functions has not been achieved to date but may be a promising strategy to prolong pulmonary allograft and recipient survival in the future.

## Author Contributions

BO drafted and wrote the manuscript. WJ drafted and wrote the manuscript. All authors contributed to the article and approved the submitted version.

## Conflict of Interest

The authors declare that the research was conducted in the absence of any commercial or financial relationships that could be construed as a potential conflict of interest.

## Publisher’s Note

All claims expressed in this article are solely those of the authors and do not necessarily represent those of their affiliated organizations, or those of the publisher, the editors and the reviewers. Any product that may be evaluated in this article, or claim that may be made by its manufacturer, is not guaranteed or endorsed by the publisher.

## References

[1] Chambers DC Yusen RD Cherikh WS Goldfarb SB Kucheryavaya AY Khusch K. The Registry of the International Society for Heart and Lung Transplantation: Thirty-Fourth Adult Lung And Heart-Lung Transplantation Report-2017; Focus Theme: Allograft Ischemic Time. J Hear Lung Transpl (2017) 36:1047–59. doi: 10.1016/j.healun.2017.07.016 28784324

[2] Levine DJ Glanville AR Aboyoun C Belperio J Benden C Berry GJ. Antibody-Mediated Rejection of the Lung: A Consensus Report of the International Society for Heart and Lung Transplantation. J Hear Lung Transpl (2016) 35:397–406. doi: 10.1016/j.healun.2016.01.1223 27044531

[3] Koenig A Thaunat O. Lymphoid Neogenesis and Tertiary Lymphoid Organs in Transplanted Organs. Front Immunol (2016) 7:1–9. doi: 10.3389/fimmu.2016.00646 28082981 PMC5186756

[4] Chong AS Khiew SH. Transplantation Tolerance: Don’t Forget About the B Cells. Clin Exp Immunol (2017) 189:171–80. doi: 10.1111/cei.12927 PMC5508319 28100001

[5] Frost AE Jammal CT Cagle PT. Hyperacute Rejection Following Lung Transplantation. Chest (1996) 110:559–62. doi: 10.1378/chest.110.2.559 8697867

[6] Cleary SJ Kwaan N Tian JJ Calabrese DR Mallavia B Magnen M. Complement Activation on Endothelium Initiates Antibody-Mediated Acute Lung Injury. J Clin Invest (2020) 130:5909–23. doi: 10.1172/JCI138136 PMC7598054 32730229

[7] Cunningham AC Zhang JG Moy JV Ali S Kirby JA. A Comparison of the Antigen-Presenting Capabilities of Class II MHC-Expressing Human Lung Epithelial and Endothelial Cells. Immunology (1997) 91:458–63. doi: 10.1046/j.1365-2567.1997.d01-2249.x PMC1364017 9301537

[8] Kreisel D Richardson SB Li W Lin X Kornfeld CG Sugimoto S. MHC Class II Expression by Pulmonary non-Hematopoietic Cells Plays a Critical Role in Controlling Local Inflammatory Responses. J Immunol (2010) 185:3809–13. doi: 10.4049/jimmunol.1000971 PMC3897247 20810992

[9] Valenzuela NM McNamara JT Reed EF. Antibody-Mediated Graft Injury: Complement-Dependent and Complement-Independent Mechanisms. Curr Opin Organ Transpl (2014) 19:33–40. doi: 10.1097/MOT.0000000000000040 PMC4080796 24316758

[10] Saini D Weber J Ramachandran S Phelan D Tiriveedhi V Lui M. Alloimmunity Induced Autoimmunity as a Potential Mechanism In The Pathogenesis of Chronic Rejection of Human Lung Allograftsa. J Heart Lung Transplant (2011) 30:624–31. doi: 10.1016/j.healun.2011.01.708 PMC3091959 21414808

[11] Fukami N Ramachandran S Saini D Walter M Chapman W Patterson GA. Antibodies to MHC Class I Induces Autoimmunity: Role in the Pathogenesis of Chronic Rejection. J Immunol (2009) 182:309–18. doi: 10.4049/jimmunol.182.1.309 PMC2802821 19109162

[12] Amara U Flierl MA Rittisch D Klos D Chen H Acker B. Molecular Intercommunication Between the Complement and Coagulation Systems. J Immunol (2010) 185:5628–36. doi: 10.4049/jimmunol.0903678 PMC3123139 20870944

[13] Trayssac M Nègre-Salvayre A Thomsen M. Mechanisms of Human Smooth Muscle Cell Proliferation and Transplant Vasculopathy Induced by HLA Class I Antibodies: *In Vitro* and *In Vivo* Studies. Hum Immunol (2012) 73:1253–60. doi: 10.1016/j.humimm.2012.06.012 22789624

[14] Kuo HH Morrell CN Baldwin WM. Alloantibody Induced Platelet Responses in Transplants: Potent Mediators in Small Packages. Hum Immunol (2012) 73:1233–8. doi: 10.1016/j.humimm.2012.06.011 PMC3496803 22789623

[15] Lee CY Lotfi-Emran S Erdinc M Murata K Velidedeoglu E Fox-Talbot K. The Involvement of FcR Mechanisms in Antibody-Mediated Rejection. Transplantation (2007) 84:1324–34. doi: 10.1097/01.tp.0000287457.54761.53 18049118

[16] Legris T Picard C Todorova D Lyonnet L Laporte C Dumoulin C. Antibody-Dependent NK Cell Activation is Associated With Late Kidney Allograft Dysfunction and the Complement-Independent Alloreactive Potential of Donor-Specific Antibodies. Front Immunol (2016) 7:288. doi: 10.3389/fimmu.2016.00288 27563301 PMC4980873

[17] Brugière O Roux A Le Pavec J Sroussi D Parquin F Pradère P. Role of C1q-Binding Anti-HLA Antibodies as a Predictor of Lung Allograft Outcome. Eur Respir J (2018) 52:1–11. doi: 10.1183/13993003.01898-2017 29976654

[18] Smith JD Ibrahim MW Newell H Danskine AJ Soresi S Burke MM. Pre-Transplant Donor HLA-Specific Antibodies: Characteristics Causing Detrimental Effects on Survival After Lung Transplantation. J Heart Lung Transpl (2014) 33:1074–82. doi: 10.1016/j.healun.2014.02.033 24954882

[19] Tague LK Witt CA Byers DE Yusen RD Aguilar PR Kulkarni HS. Association Between Allosensitization and Waiting List Outcomes Among Adult Lung Transplant Candidates in the United States. Ann Am Thorac Soc (2019) 16:846–52. doi: 10.1513/AnnalsATS.201810-713OC PMC6600838 30763122

[20] Aversa M Darley DR Hirji A Snyder L Lyu D Parquin D. Approaches to the Management of Sensitized Lung Transplant Candidates: Findings From an International Survey. J Hear Lung Transpl (2020) 39:S315. doi: 10.1016/j.healun.2020.01.709

[21] Young KA Ali HA Beermann KJ Reynolds JM Snyder lD. Lung Transplantation and the Era of the Sensitized Patient. Front Immunol (2021) 12:689420. doi: 10.3389/fimmu.2021.689420 34122454 PMC8187850

[22] Alcorn J Concept Paper: Continuous Distribution of Lungs. Concept Paper: Continuous Distribution of Lungs . Available at: https://optn.transplant.hrsa.gov/governance/public-comment/continuous-distribution-of-lungs-concept-paper/.

[23] Siu JHY Surendrakumar V Richards JA Pettigrew GJ. T Cell Allorecognition Pathways in Solid Organ Transplantation. Front Immunol (2018) 9:2548. doi: 10.3389/fimmu.2018.02548 30455697 PMC6230624

[24] Crotty S. T Follicular Helper Cell Biology: A Decade of Discovery and Diseases. Immunity (2019) 50:1132–48. doi: 10.1016/j.immuni.2019.04.011 PMC6532429 31117010

[25] Walters GD Vinuesa CG. Follicular Helper Cells in Transplantation. Transplantation (2016) 100:1650–5. doi: 10.1097/TP.0000000000001217 27362303

[26] Conlon TM Saeb-Parsy K Cole JL Motallebzadeh R Qureshi MS Rehakova S. Germinal Center Alloantibody Responses Are Mediated Exclusively by Indirect-Pathway CD4 T Follicular Helper Cells. J Immunol (2012) 188:2643–52. doi: 10.4049/jimmunol.1102830 PMC3378630 22323543

[27] Gatto D Wood K Brink R. EBI2 Operates Independently of But in Cooperation With CXCR5 and CCR7 to Direct B Cell Migration and Organization in Follicles and the Germinal Center. J Immunol (2011) 187:4621–8. doi: 10.4049/jimmunol.1101542 21948984

[28] Chong AS Sciammas R. Memory B Cells in Transplantation. Memory B Cells in Transplantation. Transplantation (2015) 99:21–8. doi: 10.1097/TP.0000000000000545 PMC4273865 25525921

[29] Viant C Wirthmiller T ElTanbouly MA Chen ST Cipolla M Ramos V. Germinal Center-Dependent and -Independent Memory B Cells Produced Throughout the Immune Response. J Exp Med (2021) 218:e20202489. doi: 10.1084/jem.20202489 34106207 PMC8193567

[30] Cancro MP Tomayko MM. Memory B Cells and Plasma Cells: The Differentiative Continuum of Humoral Immunity. Immunol Rev (2021) 303:72–82. doi: 10.1111/imr.13016 34396546

[31] Schmitz R Fitch ZW Schroder PM Choi AY Jackson AM Knechtle SJ. B Cells in Transplant Tolerance and Rejection: Friends or Foes? Transpl Int (2020) 33:30–40. doi: 10.1111/tri.13549 31705678 PMC7184555

[32] Nellore A Killian JT Porrett PM. Memory B Cells in Pregnancy Sensitization. Front Immunol (2021) 12:688987. doi: 10.3389/fimmu.2021.688987 34276679 PMC8278195

[33] Porrett PM. Biologic Mechanisms and Clinical Consequences of Pregnancy Alloimmunization. Am J Transplant (2018) 18:1059–67. doi: 10.1111/ajt.14673 29369525

[34] Hiepe F Dörner T Hauser AJ Hoyer BF Mei H Radbruch A. Long-Lived Autoreactive Plasma Cells Drive Persistent Autoimmune Inflammation. Nat Rev Rheumatol (2011) 73(7):170–8. doi: 10.1038/nrrheum.2011.1 21283146

[35] Lightman SM Utley A Lee KP. Survival of Long-Lived Plasma Cells (LLPC): Piecing Together the Puzzle. Front Immunol (2019) 10:965. doi: 10.3389/fimmu.2019.00965 31130955 PMC6510054

[36] Manz RA Löhning M Cassese G Thiel A Radbruch A. Survival of Long-Lived Plasma Cells is Independent of Antigen. Int Immunol (1998) 10:1703–11. doi: 10.1093/intimm/10.11.1703 9846699

[37] Khodadadi L Cheng Q Radbruch A Hiepe F. The Maintenance of Memory Plasma Cells. Front Immunol (2019) 10:721. doi: 10.3389/fimmu.2019.00721 31024553 PMC6464033

[38] Benet Z Jing Z Fooksman DR. Plasma Cell Dynamics in the Bone Marrow Niche. Cell Rep (2021) 34:108733. doi: 10.1016/j.celrep.2021.108733 33567286 PMC8023250

[39] Aguilar PR Carpenter D Ritter J Yusen RD Witt CA Byers DE. The Role of C4d Deposition in the Diagnosis of Antibody-Mediated Rejection After Lung Transplantation. Am J Transpl (2018) 18:936–44. doi: 10.1111/ajt.14534 PMC5878693 28992372

[40] Roux A Levine DJ Zeevi A Hachem R Halloran K Halloran PF. Banff Lung Report: Current Knowledge and Future Research Perspectives for Diagnosis and Treatment of Pulmonary Antibody-Mediated Rejection (AMR). Am J Transpl (2019) 19:21–31. doi: 10.1111/ajt.14990 29956477

[41] Cochrane AB Levine D Ponor I Philogene M Jang M Tunc I. Outcomes of ISHLT Lung Transplant AMR. J Hear Lung Transpl (2020) 39:S78. doi: 10.1016/j.healun.2020.01.1298

[42] Fukami N Ramachandran S Saini D Walter M Chapman W Patterson A. Antibodies to MHC Class I Induce Autoimmunity: Role in the Pathogenesis of Chronic Rejection. J Immunol (2009) 182:309–18. doi: 10.4049/jimmunol.182.1.309 PMC2802821 19109162

[43] Roux A Bendib Le Lan I Holifanjaniaina S Thomas KA Hamid AM Picard C. Antibody-Mediated Rejection in Lung Transplantation: Clinical Outcomes and Donor-Specific Antibody Characteristics. Am J Transpl (2016) 16:1216–28. doi: 10.1111/ajt.13589 26845386

[44] Visentin J Chartier A Massara L Linares G Guidicelli G Blanchard E. Lung Intragraft Donor-Specific Antibodies as a Risk Factor for Graft Loss. J Hear Lung Transpl (2016) 35:1418–26. doi: 10.1016/j.healun.2016.06.010 27450460

[45] Sacreas A Taupin AL Emonds MP Daniels L Van Raemdonck DE Vos R. Intragraft Donor-Specific Anti-HLA Antibodies in Phenotypes of Chronic Lung Allograft Dysfunction. Eur Respir J (2019) 54:1900847. doi: 10.1183/13993003.00847-2019 31439680

[46] Hsiao HM Li W Gelman AE Krupnick AS Kreisel D. The Role of Lymphoid Neogenesis in Allografts. Am J Transpl (2016) 16:1079–85. doi: 10.1111/ajt.13645 PMC4803576 26614734

[47] Jones GW Jones SA. Ectopic Lymphoid Follicles: Inducible Centres for Generating Antigen-Specific Immune Responses Within Tissues. Immunology (2016) 147:141–51. doi: 10.1111/imm.12554 PMC4717241 26551738

[48] Neyt K Perros F GeurtsvanKessel CH Hammad H Lambrecht BN. Tertiary Lymphoid Organs in Infection and Autoimmunity. Trends Immunol (2012) 33:297–305. doi: 10.1016/j.it.2012.04.006 22622061 PMC7106385

[49] Vandermeulen E Lammertyn E Verleden SE Ruttens D Bellon H Ricciardi M. Immunological Diversity in Phenotypes of Chronic Lung Allograft Dysfunction: A Comprehensive Immunohistochemical Analysis. Transpl Int (2017) 30:134–43. doi: 10.1111/tri.12882 27933655

[50] Alsughayyir J Pettigrew GJ Motallebzadeh R. Spoiling for a Fight: B Lymphocytes as Initiator and Effector Populations Within Tertiary Lymphoid Organs in Autoimmunity and Transplantation. Front Immunol (2017) 8. doi: 10.3389/fimmu.2017.01639 PMC5703719 29218052

[51] Thaunat O Kerjaschki D Nicoletti A. Is Defective Lymphatic Drainage a Trigger for Lymphoid Neogenesis? Trends Immunol (2006) 27:441–5. doi: 10.1016/j.it.2006.08.003 16920402

[52] Gonzalez M Mackay F Browning JL Kosco-Vilbois MH Noelle RJ. The Sequential Role of Lymphotoxin and B Cells in the Development of Splenic Follicles. J Exp Med (1998) 187:997–1007. doi: 10.1084/jem.187.7.997 9529316 PMC2212214

[53] Smirnova NF Conlon TM Morrone C Dorfmuller P Humbert M Stathopoulos GT. Inhibition of B Cell–Dependent Lymphoid Follicle Formation Prevents Lymphocytic Bronchiolitis After Lung Transplantation. JCI Insight (2019) 4:e123971. doi: 10.1172/jci.insight.123971 PMC6413786 30728330

[54] Watanabe T Martinu T Chruscinski A Boonstra K Joe B Horie M. A B Cell–Dependent Pathway Drives Chronic Lung Allograft Rejection After Ischemia–Reperfusion Injury in Mice. Am J Transpl (2019) 19:3377–89. doi: 10.1111/ajt.15550 31365766

[55] Zhang Q Reed EF. The Importance of non-HLA Antibodies in Transplantation. Nat Rev Nephrol (2016) 12:484–95. doi: 10.1038/nrneph.2016.88 PMC5669045 27345243

[56] Rao U Sharma M Mohanakumar T Ahn C Gao A Kaza V. Prevalence of Antibodies to Lung Self-Antigens (Kα1 Tubulin and Collagen V) and Donor Specific Antibodies to HLA in Lung Transplant Recipients and Implications for Lung Transplant Outcomes: Single Center Experience. Transpl Immunol (2019) 54:65–72. doi: 10.1016/j.trim.2019.02.006 30794945 PMC6513684

[57] Benichou G Alessandrini A Charrad R-S Wilkes DS. Induction of Autoimmunity After Allotransplantation. Front Biosci (2007) 12:4362–9. doi: 10.2741/2393 17485380

[58] Yarkoni Y Getahun A Cambier JC. Molecular Underpinning of B-Cell Anergy. Immunol Rev (2010) 237:249–63. doi: 10.1111/j.1600-065X.2010.00936.x PMC2968701 20727040

[59] Cook MC Basten A Fazekas De St. Groth B. Rescue of Self-Reactive B Cells by Provision of T Cell Help *In Vivo*. Eur. J Immunol (1998) 28:2549–58. doi: 10.1002/(SICI)1521-4141(199808)28:08<2549::AID-IMMU2549>3.0.CO;2-O PMC4260656 9710232

[60] Win TS Rehakova S Negus MC Saeb-Parsy K Goddard M Conlon TM. Donor CD4 T Cells Contribute to Cardiac Allograft Vasculopathy by Providing Help for Autoantibody Production. Circ Hear Fail (2009) 2:361–9. doi: 10.1161/CIRCHEARTFAILURE.108.827139 19808360

[61] Cornaby C Gibbons L Mayhew V Sloan CS Welling A Poole BD. B Cell Epitope Spreading: Mechanisms and Contribution to Autoimmune Diseases. Immunol Lett (2015) 163:56–68. doi: 10.1016/j.imlet.2014.11.001 25445494

[62] Bharat A Chiu S Zheng Z Sun H Yeldandi A DeCamp MM. Lung-Restricted Antibodies Mediate Primary Graft Dysfunction and Prevent Allotolerance After Murine Lung Transplantation. Am J Respir Cell Mol Biol (2016) 55:532–41. doi: 10.1165/rcmb.2016-0077OC PMC5070112 27144500

[63] Zeng Q Ng Y-H Singh T Jiang K Sheriff KA Ippolito R. B Cells Mediate Chronic Allograft Rejection Independently of Antibody Production. J Clin Invest (2014) 124:1052–6. doi: 10.1172/JCI70084 PMC3934170 24509079

[64] Sanderson NSR Zimmermann M Eilinger L Gubser C Schaeren-Wiemers N Lindberg RLP. Cocapture of Cognate and Bystander Antigens can Activate Autoreactive B Cells. Proc Natl Acad Sci U S A (2017) 114:734–9. doi: 10.1073/pnas.1614472114 PMC5278454 28057865

[65] Panda S Ding JL. Natural Antibodies Bridge Innate and Adaptive Immunity. J Immunol (2015) 194:13–20. doi: 10.4049/jimmunol.1400844 25527792

[66] See SB Aubert O Loupy A Veras Y Lebreton X Gao B. Post-Transplant Natural Antibodies Associate With Kidney Allograft Injury and Reduced Long-Term Survival. J Am Soc Nephrol (2018) 29:1761–70. doi: 10.1681/ASN.2017111157 PMC6054349 29602833

[67] Rolán HC Xavier MN Santos RL Tsolis RM. Natural Antibody Contributes to Host Defense Against an Attenuated Brucella Abortus virB Mutant. Infect Immun (2009) 77:3004–13. doi: 10.1128/IAI.01114-08 PMC2708545 19364836

[68] Reyneveld G Savelkoul HFJ. And Parmentier, H Current Understanding of Natural Antibodies and Exploring the Possibilities of Modulation Using Veterinary Models K. A Review Front Immunol (2020) 11:1–19. doi: 10.3389/fimmu.2020.02139 PMC7511776 33013904

[69] Gao B Rong C Procheray F Moore C Girouard TC Saidman SL. Evidence to Support a Contribution of Polyreactive Antibodies to HLA Serum Reactivity. Transplantation (2016) 100:217–26. doi: 10.1097/TP.0000000000000840 PMC5384715 26285015

[70] Gao B Moore C Porcheray F Rong C Abidoglu C DeVito J. Pre-Transplant IgG Reactivity to Apoptotic Cells Correlates With Late Kidney Allograft Loss. Am J Transpl (2014) 14:1581–91. doi: 10.1111/ajt.12763 PMC4120834 24935695

[71] Porcheray F Fraser JW Gao B McColl A DeVito J Dargon I. Polyreactive Antibodies Developing Amidst Humoral Rejection of Human Kidney Grafts Bind Apoptotic Cells and Activate Complement. Am J Transpl (2013) 13:2590–600. doi: 10.1111/ajt.12394 PMC3864117 23919437

[72] Budding K van de Graaf EA Kardol-Hoefnagel T Oudijk EJD Kwakkel-vanErp JM Hack CE. Antibodies Against Apoptotic Cells Present in End-Stage Lung Disease Patients do Not Correlate With Clinical Outcome After Lung Transplantation. Front Immunol (2017) 8:1–10. doi: 10.3389/fimmu.2017.00322 28377770 PMC5359236

[73] Noorchashm H Reed AJ Rostami SY Mozaffari R Zekavat G Koeberlein B. B Cell-Mediated Antigen Presentation is Required for the Pathogenesis of Acute Cardiac Allograft Rejection. J Immunol (2006) 177:7715–22. doi: 10.4049/jimmunol.177.11.7715 17114442

[74] Chong AS. B Cells as Antigen-Presenting Cells in Transplantation Rejection and Tolerance. B Cells as Antigen-Presenting Cells in Transplantation Rejection and Tolerance. Cell Immunol (2020) 349:104061. doi: 10.1016/j.cellimm.2020.104061 32059816 PMC7210794

[75] Wortel CM Heidt S. Regulatory B Cells: Phenotype, Function and Role in Transplantation. Transpl Immunol (2017) 41:1–9. doi: 10.1016/j.trim.2017.02.004 28257995

[76] Alhabbab RY Nova-Lamperti E Aravena O Burton HM Lechler RI Dorling A. Regulatory B Cells: Development, Phenotypes, Functions, and Role in Transplantation. Immunol Rev (2019) 292:164–79. doi: 10.1111/imr.12800 31559645

[77] Salama AD Remuzzi G Harmon WE Sayegh MH. Challenges to Achieving Clinical Transplantation Tolerance. J Clin Invest (2001) 108:943. doi: 10.1172/JCI200114142 11581293 PMC200962

[78] Chesneau M Danger R Soulillou JP Brouard S. B Cells in Operational Tolerance. Hum Immunol (2018) 79:373–9. doi: 10.1016/j.humimm.2018.02.009 29458071

[79] Newell KA Adams AB Turka LA. Biomarkers of Operational Tolerance Following Kidney Transplantation – The Immune Tolerance Network Studies of Spontaneously Tolerant Kidney Transplant Recipients. Hum Immunol (2018) 79:380. doi: 10.1016/j.humimm.2018.02.007 29448053 PMC5924709

[80] Piloni D Morosini M Magni S Balderacchi A Inghilleri S Cova E. Peripheral CD19+CD24highCD38high B-Regulatory Cells in Lung Transplant Recipients. Transpl Immunol (2019) 57:101245. doi: 10.1016/j.trim.2019.101245 31526864

[81] Zhao Y Gillen JR Meher AK Burns JA Kron IL Lau CL. Rapamycin Prevents Bronchiolitis Obliterans Through Increasing Regulatory B Cells Infiltration in a Murine Tracheal Transplantation Model. J Thorac Cardiovasc Surg (2016) 151:487–96.e3. doi: 10.1016/j.jtcvs.2015.08.116 26481278 PMC4728002

[82] Li J Luo Y Wang X Feng G. Regulatory B Cells and Advances in Transplantation. J Leukoc Biol (2019) 105:657–68. doi: 10.1002/JLB.5RU0518-199R 30548970

[83] Hirose T Tanaka Y Tanaka A Sakai H Sasaki Y Shinohara N. PD-L1/PD-L2-Expressing B-1 Cells Inhibit Alloreactive T Cells in Mice. PloS One (2017) 12:e0178765. doi: 10.1371/journal.pone.0178765 28570665 PMC5453578

[84] Xu G Shi Y. Apoptosis Signaling Pathways and Lymphocyte Homeostasis. Cell Res (2007) 17:759–71. doi: 10.1038/cr.2007.52 17576411

[85] Collison LW Chaturvedi V Henderson AL Giacomin PR Guy C Bankoti J. IL-35-Mediated Induction of a Potent Regulatory T Cell Population. Nat Immunol (2010) 11:1093–101. doi: 10.1038/ni.1952 PMC3008395 20953201

[86] Hagn M Jahrsdörfer B. Why do Human B Cells Secrete Granzyme B? Insights Into a Novel B-Cell Differentiation Pathway. Oncoimmunology (2012) 1:1368. doi: 10.4161/onci.22354 23243600 PMC3518509

[87] Mielle J Audo R Hahne M Macia L Combe B Morel J. IL-10 Producing B Cells Ability to Induce Regulatory T Cells is Maintained in Rheumatoid Arthritis. Front Immunol (2018) 9:961. doi: 10.3389/fimmu.2018.00961 29774031 PMC5943500

[88] Clatworthy MR Watson CJE Plotnek G Bardsley V Chaudhry AN Bradley JA. B-Cell–Depleting Induction Therapy and Acute Cellular Rejection. N Engl J Med (2009) 360:2683. doi: 10.1056/NEJMc0808481 19535812 PMC4143588

[89] Choi AY Manook M Olaso D Ezekian B Park J Freischlag K. Emerging New Approaches in Desensitization: Targeted Therapies for HLA Sensitization. Front Immunol (2021) 12:2219. doi: 10.3389/fimmu.2021.694763 PMC8226120 34177960

[90] Yamada C Ramon DS Cascalho M Sung RS Leichtman AB Samaniego M. Efficacy of Plasmapheresis on Donor-Specific Antibody Reduction by HLA Specificity in Post–Kidney Transplant Recipients. Transfusion (2015) 55:727. doi: 10.1111/trf.12923 25385678 PMC4911015

[91] Muller YD Aubert JD Vionnet J Rotman S Sadallah S Aubert V. Acute Antibody-Mediated Rejection 1 Week After Lung Transplantation Successfully Treated With Eculizumab, Intravenous Immunoglobulins, and Rituximab. Transplantation (2018) 102:e301–3. doi: 10.1097/TP.0000000000002165 29521880

[92] Dawson KL Parulekar A Seethamraju H. Treatment of Hyperacute Antibody-Mediated Lung Allograft Rejection With Eculizumab. J Heart Lung Transpl (2012) 31:1325–6. doi: 10.1016/j.healun.2012.09.016 23063321

[93] Parquin F Cuquemelle E Camps E Devaquet J Houllbracq MP Sage E. C1-Esterase Inhibitor Treatment for Antibody-Mediated Rejection After Lung Transplantation: Two Case Reports. Eur Respir J (2020) 55:1902027. doi: 10.1183/13993003.02027-2019 32079639

[94] Thomas KA Valenzuela NM Gjertson D Mulder A Fishbein MC Parry GC. An Anti-C1s Monoclonal, TNT003, Inhibits Complement Activation Induced by Antibodies Against HLA. Am J Transplant (2015) 15:2037–49. doi: 10.1111/ajt.13273 PMC4654252 25904443

[95] Mühlbacher J Jilma B Wahrmann M Bartko J Eskandary F Schörgenhofer C. Blockade of HLA Antibody-Triggered Classical Complement Activation in Sera From Subjects Dosed With the Anti-C1s Monoclonal Antibody TNT009-Results From a Randomized First-In-Human Phase 1 Trial. Transplantation (2017) 101:2410–8. doi: 10.1097/TP.0000000000001804 PMC5610566 28926521

[96] Eskandary F Jilma B Mühlbacher J Wahrmann M Regele H Kozakowski N. Anti-C1s Monoclonal Antibody BIVV009 in Late Antibody-Mediated Kidney Allograft Rejection-Results From a First-in-Patient Phase 1 Trial. Am J Transplant (2018) 18:916–26. doi: 10.1111/ajt.14528 28980446

[97] Jordan SC Lorant T Choi J Kjellman C Winstedt L Bengtsson M. IgG Endopeptidase in Highly Sensitized Patients Undergoing Transplantation. N Engl J Med (2017) 377:442–53. doi: 10.1056/NEJMoa1612567 28767349

[98] Kazatchkine MD Kaveri SV. Immunomodulation of Autoimmune and Inflammatory Diseases With Intravenous Immune Globulin. N Engl J Med (2001) 345:747–55. doi: 10.1056/NEJMra993360 11547745

[99] Levine MH Abt PL. Treatment Options and Strategies for Antibody Mediated Rejection After Renal Transplantation. Semin Immunol (2012) 24:136. doi: 10.1016/j.smim.2011.08.015 21940179 PMC3248625

[100] Benedetti EC Chery G Hartwig M Hulbert A Reynolds J Snyder L. Intravenous Immunoglobulin in Sensitized Lung Transplant Recipients and Early Outcomes. J Hear Lung Transpl (2016) 35:S237–8. doi: 10.1016/j.healun.2016.01.673

[101] Snyder LD Gray AL Reynolds JM Arepally GM Bedoya A Hartwig MG. Antibody Desensitization Therapy in Highly Sensitized Lung Transplant Candidates. Am J Transpl (2014) 14:849. doi: 10.1111/ajt.12636 PMC4336170 24666831

[102] Ionescu L Urschel S. Memory B Cells and Long-Lived Plasma Cells. Transplantation (2019) 103:890–8. doi: 10.1097/TP.0000000000002594 30747835

[103] Hachem RR Yusen RD Meyers BF Aloush AA Mohanakumar T Patterson GA. Anti-Human Leukocyte Antigen Antibodies and Preemptive Antibody-Directed Therapy After Lung Transplantation. J Heart Lung Transpl (2010) 29:973–80. doi: 10.1016/j.healun.2010.05.006 PMC2926224 20558084

[104] Frampton JE. Inebilizumab: First Approval. Drugs (2020) 80:1259–64. doi: 10.1007/s40265-020-01370-4 PMC7387876 32729016

[105] Bhoj VG Arhontoulis D Wertheim G Capobianchi J Callahan CA Ellebrecht CT. Persistence of Long-Lived Plasma Cells and Humoral Immunity in Individuals Responding to CD19-Directed CAR T-Cell Therapy. Blood (2016) 128:360–70. doi: 10.1182/blood-2016-01-694356 PMC4957161 27166358

[106] Stuckey LJ Kamoun M Chan KM. Lung Transplantation Across Donor-Specific Anti-Human Leukocyte Antigen Antibodies: Utility of Bortezomib Therapy in Early Graft Dysfunction. Ann Pharmacother (2012) 46:e2. doi: 10.1345/aph.1Q509 22202499

[107] Vacha M Chery G Hulbert A Byrns J Benedetti C Copeland CAF. Antibody Depletion Strategy for the Treatment of Suspected Antibody-Mediated Rejection in Lung Transplant Recipients: Does it Work? Clin Transpl (2017) 31:e12886. doi: 10.1111/ctr.12886 27988971

[108] Frandsen EL Otero J Rutledge JC Kemna MS Albers EL Hong BJ. A Fatal Case of Bortezomib-Induced Lung Toxicity in a Young Adult Heart Transplant Recipient. Pediatr Transpl (2020) 24:e13628. doi: 10.1111/petr.13628 31815325

[109] Schroeder MA Rettig MP Lopez S Christ S Fiala M Eades W. Mobilization of Allogeneic Peripheral Blood Stem Cell Donors With Intravenous Plerixafor Mobilizes a Unique Graft. Blood (2017) 129:2680. doi: 10.1182/blood-2016-09-739722 28292947 PMC5428459

[110] Liu CL Lyle MJ Shin SC Miao CH. Strategies to Target Long-Lived Plasma Cells for Treating Hemophilia A Inhibitors. Cell Immunol (2016) 301:65–73. doi: 10.1016/j.cellimm.2016.01.005 26877251 PMC4844017

[111] Moore N Moreno Gonzales M Bonner K Smith B Park W Stegall M. Impact of CXCR4/CXCL12 Blockade on Normal Plasma Cells *In Vivo*. Am J Transpl (2017) 17:1663–9. doi: 10.1111/ajt.14236 28235241

[112] Kwun J Matignon M Manook M Guendouz S Audard V Kheav D. Daratumumab in Sensitized Kidney Transplantation: Potentials and Limitations of Experimental and Clinical Use. J Am Soc Nephrol (2019) 30:1206–19. doi: 10.1681/ASN.2018121254 PMC6622431 31227636

[113] Agarwal D Allman D Naji A. Novel Therapeutic Opportunities Afforded by Plasma Cell Biology in Transplantation. Am J Transpl (2020) 20:1984–91. doi: 10.1111/ajt.15813 32034987

[114] Wilson NA Bath NM Verhoven BM Ding X Boldt BA Sukhwal A. APRIL/BLyS Blockade Reduces Donor-Specific Antibodies in Allosensitized Mice. Transplantation (2019) 103:1372–84. doi: 10.1097/TP.0000000000002686 PMC6594891 30830041

[115] Kwun J Page E Hong JJ Gibby A Yoon J Farris AB. Neutralizing BAFF/APRIL With Atacicept Prevents Early DSA Formation and AMR Development in T Cell Depletion Induced Nonhuman Primate AMR Model. Am J Transpl (2015) 15:815–22. doi: 10.1111/ajt.13045 PMC5504528 25675879

[116] Banham GD Flint SM Torpey N Lyons PA Shanahan DN Gibson A. Belimumab in Kidney Transplantation: An Experimental Medicine, Randomised, Placebo-Controlled Phase 2 Trial. Lancet (2018) 391:2619–30. doi: 10.1016/S0140-6736(18)30984-X PMC7617033 29910042

[117] Mosharmovahed B Fatahi Y Mohebbi B Ghorbanian SA Assadiasl S. Tocilizumab in Transplantation. Eur J Clin Pharmacol (2020) 76:765–73. doi: 10.1007/s00228-020-02864-6 32266480

[118] Roll P Muhammad K Schumann M Kleinert S Einsele H Dörner T. *In Vivo* Effects of the Anti–Interleukin-6 Receptor Inhibitor Tocilizumab on the B Cell Compartment. Arthritis Rheumatol (2011) 63:1255–64. doi: 10.1002/art.30242 21305508

[119] Kopf M Herren S Wiles MV Pepys MB Kosco-Vilbois MH. Interleukin 6 Influences Germinal Center Development and Antibody Production *via* a Contribution of C3 Complement Component. J Exp Med (1998) 188:1895–906. doi: 10.1084/jem.188.10.1895 PMC2212418 9815267

[120] Verleden SE Martens A Vandermeulen E Bellon H Heigl T Sacreas A. The Association of IL-6 and IL-8 Within 72 Hours Post-Transplant and Short and Long Term Outcome. J Hear Lung Transpl (2017) 36:S187. doi: 10.1016/j.healun.2017.01.492

[121] Ross DJ Der Hovanessian A Kubak B Reed E Natori C Schaenman J. Combination Therapies Including Tocilizumab Decrease the Progression of CLAD: Initial Clinical Experience. J Hear Lung Transpl (2019) 38:S405. doi: 10.1016/j.healun.2019.01.1032

[122] Palmer SM Miralles AP Lawrence CM Gaynor JW Davis RD Tapson VF. Rabbit Antithymocyte Globulin Decreases Acute Rejection After Lung Transplantation: Results of a Randomized, Prospective Study. Chest (1999) 116:127–33. doi: 10.1378/chest.116.1.127 10424515

[123] January SE Fester KA Bain KB Kulkarni HS Witt CA Byers DE. Rabbit Antithymocyte Globulin for the Treatment of Chronic Lung Allograft Dysfunction. Clin Transpl (2019) 33:e13708. doi: 10.1111/ctr.13708 31494969

[124] Ensor CR Goehring KC Iasella CJ Moore CA Lendermon EA McDyer JF. Belatacept for Maintenance Immunosuppression in Cardiothoracic Transplantation: The Potential Frontier. Clin Transpl (2018) 32:e13363. doi: 10.1111/ctr.13363 30058177

[125] Bray RA Gebel HM Townsend R Roberts ME Polinsky M Yang L. *De Novo* Donor-Specific Antibodies in Belatacept-Treated vs Cyclosporine-Treated Kidney-Transplant Recipients: *Post Hoc* Analyses of the Randomized Phase III BENEFIT and BENEFIT-EXT Studies. Am J Transpl (2018) 18:1783–9. doi: 10.1111/ajt.14721 PMC6055714 29509295

[126] Iasella CJ Winstead RJ Moore CA Johnson BA Feinberg AT Morrell MR. Maintenance Belatacept-Based Immunosuppression in Lung Transplantation Recipients Who Failed Calcineurin Inhibitors. Transplantation (2018) 102:171–7. doi: 10.1097/TP.0000000000001873 28691954

